# Exploratory study on tissue ablation with cryoelectrolysis

**DOI:** 10.1371/journal.pone.0283793

**Published:** 2023-04-05

**Authors:** Franco Lugnani, Jianfei Ye, Ling Yuan, John G. J. Zhao, Diana Zhang, Boris Rubinsky

**Affiliations:** 1 Hippocrates D.O.O, Divaca, Slovenia; 2 Department of Surgery, Tianjin Haibin People’s Hospital, Tianjin, China; 3 Tianjin Institute of Medical Science, Tianjin, China; 4 Medinux Tianjin Technologies Co., Ltd., Tianjin, China; 5 Department of Bioengineering, University of California Berkeley, Berkeley, CA, United States of America; Cardiff Metropolitan University, UNITED KINGDOM

## Abstract

This is an exploratory study on the effect of electrolysis, delivered during the thawing stage of a cryoablation protocol, on tissue ablation. This treatment protocol, that combines freezing and electrolysis, is named “cryoelectrolysis”. In cryoelectrolysis the cryoablation probe is also used as the electrolysis delivering electrode. The study was performed on the liver of Landrace pigs and the tissues were examined 24 hours after treatment (two pigs) and 48 hours after treatment (one pig). The cryoelectrolysis device and different cryoelectrolysis ablation configurations tested are described. This exploratory, non-statistical study shows that the addition of electrolysis expands the ablated area in comparison to cryoablation alone and that there is a substantial difference between the histological appearance of tissue treated by cryoablation alone, tissue treated by cryoablation and electrolysis at the anode and tissue treated by cryoablation and electrolysis at the cathode.

## Introduction

Cryoablation is a minimally invasive surgical procedure, that employs freezing to ablate undesirable tissues [1–3]. Cryoablation is done with a cryoablation probe, which is a thin hollow needle, cooled internally with a cryogen. The cryoprobe is inserted into the core of the tumor. As the probe begins to cool, the margin of the frozen lesion propagates in time, from the cryogen cooled cryoablation probe outward, to encompass the undesirable targeted tissue, to its outer margin [4,5]. Cryoablation has become a well-established minimally invasive tissue ablation technique [6–9]. Cryoablation has many attractive attributes. It employs simple needle-like cryoprobes that are inserted into the core of the volume targeted for ablation. The entire surgical procedure can be monitored with intraoperative medical imaging. Many of the conventional intraoperative imaging technologies such as ultrasound [10,11], MRI [12], CT and Electrical Impedance Tomography (EIT) [13] can be used. Usually, it yields centimeter size (typically up to 4 cm diameter) ablation lesions [7,14]. An often mentioned disadvantage of cryoablation is that frozen cells can survive freezing, in particular on the outer margin of the frozen lesion, where the tissue is at high subfreezing temperatures [6]. In a desire to extend the ablation region clinical cryoablation protocols employ two or more cryoablation freeze/thaw cycles in the hope that all the frozen cells, to the margin of the frozen lesion are ablated. This substantially prolongs the clinical procedure. Attempts are made to find new ways for complete ablation of all the frozen cells with a single freeze/thaw cycle [7]. The study in this paper deals with a new technique for frozen cell ablation that combines freezing and thawing with electrolysis (cryoelectrolysis).

Briefly, electrolysis is a physical phenomenon in which low mA currents delivered between two different polarity electrodes, immersed in an ionic solution, generate new chemical species and changes in pH at the electrodes. The products of electrolysis diffuse from the probes outward into the surrounding medium. The products of electrolysis are toxic to cells [15]. The toxicity depends on the concentration and the time of exposure of cells to the products of electrolysis. Studies show that the primary mechanisms that affect cell viability in electrolysis are changes in pH, due to the diffusion of the hydrogen and OH^-^ ions [16]. At the anode side a pH lower than 4.6 and at the cathode side a pH higher than 10.4, cause cell death [16]. Tissue ablation by electrolysis is used in medicine since the middle of the 19^th^ century [17]. Numerous studies were published on tissue ablation by electrolysis, e.g. [18,19–23]. Clinical electrolytic ablation is done using two electrodes inserted in the core of undesirable tissue. As electric currents flows between the electrodes, the products of electrolysis diffuse outward from the electrode. The clinical goal of the process of electrolysis is to provide electric current for a sufficiently long period of time so that the products of electrolysis diffuse from the electrode at a quantity that is sufficient to cause cell death to the outer margin of the tissue targeted for electrolytic ablation. Hundreds of patients were successfully treated with electrolysis in China and Scandinavian countries [23]. In clinic, the technology is known as Electro Chemo Therapy or EchT and has been used primarily in the liver, kidney and lung. However, EChT has not penetrated mainstream tissue ablation surgery. One of the drawbacks of electrolysis is that high concentrations of products of electrolysis and long periods of exposure to these concentrations are needed for ablation of cells with an intact membrane. To ablate the entire volume of a region targeted for ablation, the products of electrolysis must diffuse from the electrode, usually placed in the core of the targeted volume, to the outer margin of that volume. The products of electrolysis must reach a toxic level of concentration for prolonged periods of time. Typically a single clinical electrolysis ablation procedure is tens of minutes to hours long [18–20,23]. This is much longer than other ablations procedures using microwave, ultrasound, radiofrequency, electroporation and cryoablation and is probably one of the main reasons why electrolysis has not penetrated mainstream interventional surgery.

The idea of combining electrolysis and cryosurgery was inspired by the observation that electrolysis enhances cell death when the cell membrane is permeabilized by electroporation [24] and by the observation that freezing and low temperatures also permeabilize the cell membrane. Temperatures below 18°C induce lipid phase transition and permeabilize the cell membrane [25]. Freezing and thawing also increase the permeability of the cell membrane [26]. This effect of freezing is used, for example, to determine the topology of intracellular proteins [27] and in electrical impedance tomography [13]. The above observations suggested that the combination of freezing and electrolysis may also enhance cell death, in a similar way to the combination electroporation and electrolysis.

While the membrane of intact cells is mostly impermeable to products of electrolysis, the products of electrolysis could transverse cold, freezing and thawing permeabilized cell membranes. In this way the products of electrolysis can cause cell death, at a much lower concentration than the concentration required to ablate intact cells. Furthermore, if the products of electrolysis would enter every tissue treated by freezing and thawing, they could cause the death of cells which survive freezing, in particular on the outer margin of the frozen lesion at high subfreezing temperatures.

In designing cryoelectroporation devices, advantage can be taken of the metallic material of which cryosurgery probes are made. Because cryoablation probes are metallic they can also serve as the electrolysis electrode in a cryoelectrolysis protocol. Since in electrolysis, current flows between two electrodes with different polarity, cryoelectrolysis requires two electrodes. Several cryoelectrolysis probe configurations are possible. These include: two cryoelectrolysis probes, each with a different polarity, inserted in parallel in the tissue targeted for ablation; one cryoelectrolysis probe inserted in the tissue targeted for ablation and a surface pad with a different polarity; different polarity cryoelectrolysis ablation probes inserted in several distant tumors.

Several studies were published on the concept of cryoelectrolysis [28–32]. First, a study was performed to determine if products of electrolysis can diffuse through a frozen media. Experiments with pH dyes in frozen gels, have shown that products of electrolysis can diffuse from the electrolysis electrode through the frozen media, at high subfreezing temperatures [29]. A subsequent study [30], simulated a cryoelectrolysis procedure in a gel. It was found that the products of electrolysis diffuse from the cryoelectrolysis probe (a conventional cryosurgery probe in which the metal body served also as the electrolysis electrode) outward during the early stages of freezing and throughout the thawing period. During thawing the products of electrolysis diffuse to the outer margin of the frozen region. It was surprising to find that the diffusion of products of electrolysis starts already at the onset of thawing as soon as freezing concludes. This can be explained by the way in which tissue thaws [31]. Upon thawing the temperature of the frozen lesion elevates rapidly to the change of phase temperature, and remains at that temperature throughout the thawing process. Experiments with cryoelectrolysis in gels were followed with experiments with fresh, *in vitro* pig liver [32]. Cryoelectrolysis experiments were performed with two cryoelectrolysis probes (probes in which the cryosurgery probes were also used as the electrolysis electrodes) inserted in the liver tissue in parallel. Histological analysis of liver treated by a combination freezing/thawing and electrolysis shows that the cells have a different appearance from cells in liver treated by freezing/thawing alone. The main difference is the appearance of the nucleus. This supports the suggestion that the products of electrolysis enter and affect the interior of the frozen and thawed cells. This study was followed by an acute single animal study on cryoelectrolysis in the liver. In that study a comparison was made between cells ablated by cryosurgery alone, electrolysis alone and a combination cryosurgery and electrolysis (cryoelectrolysis) [33]. Histology done after 3h survival shows that the mixed rim of live and dead cells found around the ablated lesion in both cryosurgery and electrolytic ablation is replaced by a sharp margin between life and dead cells in cryoelectrolysis. The appearance of the dead cells in each, cryoelectrolysis, cryosurgery and electrolytic ablation is different. The previous animal studies show that there is a synergistic effect of freezing/thawing and electrolysis. These studies have also verified that it convenient to use cryoablation probes as the electrolysis electrodes.

Our previous cryoelectrolysis studies were done in gels, liver tissue in vitro and acute liver tissue in vivo. This is an exploratory, non-statistical study which expands on the previous studies and is the first *in vivo* cryoelectrolysis liver ablation study with 24 hours and 48 hours follow up. It compares the outcome of a cryoelectrolysis protocol, with the outcome of a typical single freeze-thaw cryoablation protocol.

It is well established that tissue ablation by electrolysis alone is a lengthy, hours long procedure [19–23], while cryosurgery is much shorter procedure, several tens of minutes long. This is one of the main reasons why tissue ablation by electrolysis has not gained the level of clinical acceptance that cryoablation has. Because it is established that cryoablation has more clinical appeal than electrolysis, we chose to only compared cryoelectrolysis with cryoablation.

## Materials and methods

### Animal model

Experiments were conducted in compliance with the ethical and legal framework imposed by the national legislation of China. The experimental protocol was approved by the Ethics Committee of TEDA International Cardiovascular Hospital (License No.: SYXK (Jin) 2016–0004) in January 2020. A prior approval under the Welfare Ethics Review (No.: TICH-JY-20200114-1) was obtained. Subjects of the study were three 80~90 kg male Landrace pigs, supplied by Tianjin Bainong Laboratory Animal Breeding Technology Co., Ltd. with license No.: SCXK (Jin) 2015–0002. After being fasted for 24 hours, each animal was pre-medicated with a combination of xylazine hydrochloride (600mg) and Midazonlam (20mg) via intramuscular injection. Anesthesia was intravenously administered in form of Propofol (350mg - 450mg/h) and adjusted according to the heart rate. The first dose of 50μg fentanyl was used for pain relief and 30μg/h for maintenance. The pain relief was continuously delivered for 4 hours after the surgery. Access to the upper abdomen was obtained using a Chevron-like subcostal incision extended to the left side. Liver was then exposed and mobilized cutting the falciform ligaments in order to get access to the dome of the organ and to all lobes. The experiments were conducted using custom-made dedicated sterile cryoelectrolysis probes (CryoE probes, Medinux, Tianjin, China). The cryoelectrolysis probe is connected to both a source of cold and a source of electricity, which can be activated either simultaneously or individually. Each cryo-electrode placement was recorded using conventional photo/video cameras and ultrasound imaging (HITACHI-ALOKA, Model: IP-1223 DV). The surgical window was closed after completion of the test protocols and the animals were kept alive for 24 hours (two pigs), and 48 hours (one pig) with the pain prevention protocol described above.

### Cryoelectrolysis probe and cryoelectrolysis generator

**Fig 1:**
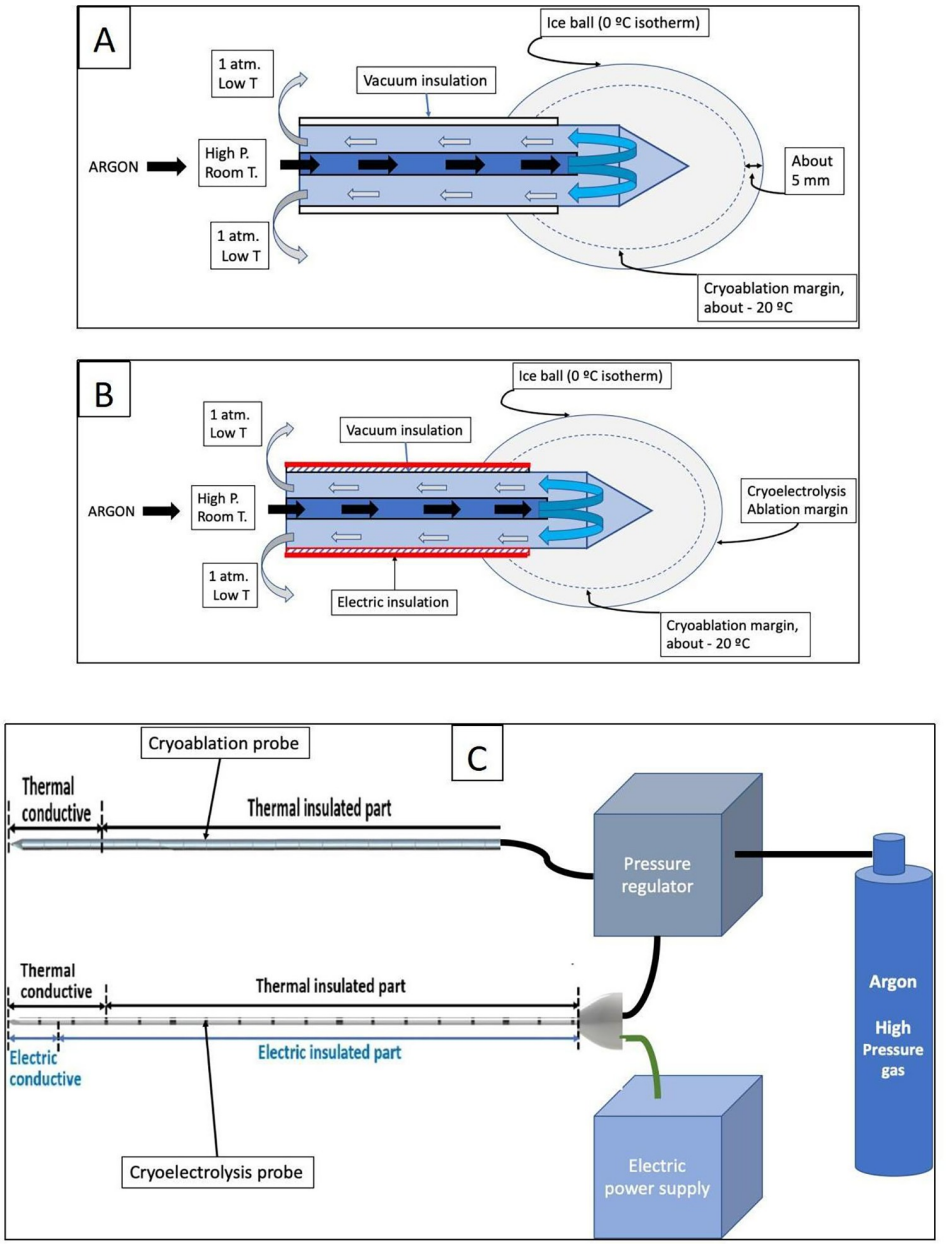
Cryoelectrolysis devices.

A prototype cryoelectrolysis device and probes have been used to carry out the experiments (CryoE Knife, model CE-80, Medinux Tianjin, China). The cryoprobe used in this study is a conventional high-pressure Argon based probe that generates low temperatures by expansion of the pressurized Argon through a Joule-Thomson orifice. A detailed description of such a probe can be found in many publications, e.g. [34]. A schematic is shown in Fig 1A. The cryoprobe is operated from a high-pressure Argon container, connected through a pressure regulator (see Fig 1C). The high-pressure, room temperature argon gas is forced through a narrow tube, a throttle with a narrow opening, and allowed to rapidly expand to atmospheric pressure in the inner chamber of the cryoablation probe (see Fig 1A and 1B). The rapid expansion of the argon causes a decrease in the temperature of the gas (the Joule-Thompson effect). Heat is withdrawn from the metallic walls of the cryoprobe, by convection and conduction. The depressurized gas is vented back through the anulus that forms between the inner narrow tube and the outer tube of the cryoprobe (Fig 1A and 1B). A vacuum insulate tube is set around the cryoprobe to facilitate precise delivery of the cold from the gas expansion chamber. Freezing begins from the cryoprobe cooling chamber outward, resulting in a frozen lesion. As illustrated by Fig 1A and 1B, there is a margin of cells which survive freezing, between the outer edge of the frozen lesion at 0°C and about– 20°C isotherm. Warming of the cryoprobe and thawing of the frozen tissue is achieved by feeding the cryoprobe with helium gas, whose rapid expansion into the inner chamber of the probe will produce heat (the Joule-Thompson effect). Cryoablation probes are usually made of metal to withstand thermal stresses and internal pressures. However, the metallic cryoablation probes also conduct electricity and therefore can be also used as electrolysis electrodes (Fig 1B). Cryoelectrolysis operates by generating products of electrolysis from the outer surface of the cryoprobe, within the frozen lesion. For precise delivery of the products of electrolysis the cryoelectrolysis electrode can be made identical to the cryoablation probe (Fig 1A), with the addition of an insulation layer along the shaft, to force the current to be delivered from the tip of the cryoprobe. The combination of freezing and electrolysis should ablate treated tissue to the margin of the frozen lesion (see Fig 1B). The electrolysis generating current is delivered through a DC power supply connected to the metal part of the conventional cryoablation probe (Fig 1C).

Conventionally, the cryoablation probe is made of a thermally insulated part and a thermally conductive part, where cooling is delivered. The cryoelectrolysis probe is also made of two parts, but one part is both thermally and electrically conductive and the other is thermally and conductive insulated. The cryoelectrolysis probes used in these experiments were 2.8 mm in diameter. The exposed electrically and thermally conductive parts were 15 mm from the tip (Fig 1C). Freezing was generated using a computerized console that can deliver a constant flow of Argon Gas at 3000 psi (Fig 1C). The Argon gas flows through an insulated hose to the cryoablation probe where a typical Joule Thomson expansion system generates the necessary cryogenic temperatures (−185.8°C). The cryoelectrolysis probe is vacuum insulated along the shaft. The cryoelectrolysis probe is also connected to a DC power supply designed to supply 50 mA per pair of electrodes at 36 V. Electrical insulation was achieved by coating the exterior of the cryoelectrolysis probe with Teflon, to the 15 mm from the tip thermally and electrically conductive part (Fig 1C).

### Experimental protocol

**Fig 2:**
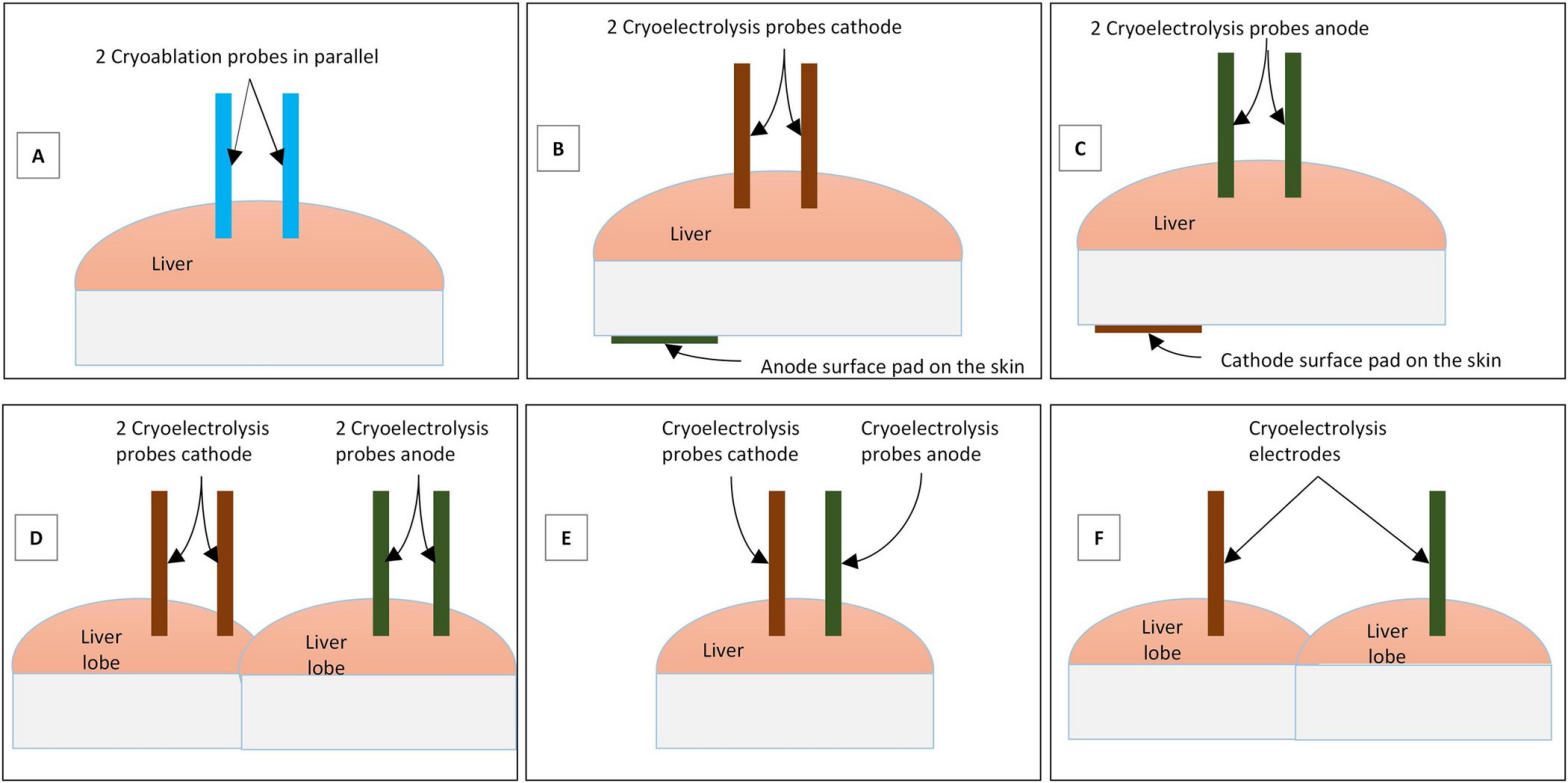
Cryoelectrodes configuration.

While cryoablation can be done with only one cryoablation probe, cryoelectrolysis requires the use of two electrodes. To be able to compare the outcome of different cryoelectrolysis treatment protocols we have used, in all the experiments, two cryoelectrolysis probes inserted in parallel perpendicular to the surface of the liver lobe with a distance of 25 mm between the probes and an insertion depth of 15 mm. An insertion template served as the probe holder. The insertion template is a 10 mm thick Teflon plate in which two 2.8 mm holes were drilled normal to the surface of the plate with 25 mm center to center apart. The insertion template was placed on the surface of the liver and the probes were inserted through the holes, perpendicular to the surface of the liver lobe. Fig 2 illustrates the different configuration used in this study. A primary purpose of the study was to find out if it is possible to use for cryoelectrolysis ablation the different configurations of electrodes in Fig 2 and, if there is a difference between the outcome of the cryoelectrolysis ablation for the different configurations, when the same current is delivered between each electrode pair. Following is a list of the configurations examined in this study. Fig 2A shows two cryoablation probes inserted in parallel to each other in one liver lobe, to provide cryoablation only controls. Fig 2B shows two cryoelectrodes inserted in the liver in parallel acting as cathodes and a surface pad on the pig thigh acting as the anode. Fig 2C shows two cryoelectrodes inserted in the liver in parallel acting as anodes and a surface pad on the pig tight acting as a cathode. Fig 2D shows two cryoelectrodes of one polarity inserted in one liver lobe in parallel, acting as cathodes; and two cryoelectrodes of another polarity inserted in another liver lobe in parallel, acting as anodes. Fig 2E shows two cryoelectrodes inserted in the liver in parallel, one acting as the cathode and the other as the anode. Fig 2F shows two cryoelectrodes inserted in two liver lobes, one acting as the cathode and the other as the anode. Table 1 provides details of the different experiments performed in this study, in the context of the schematics in Fig 2. The table shows that in all the configurations, the current through each pair of electrode cathode is 50 mA. Note that the amount of electrolytic products generated at the electrodes depends only on the current.

**Table 1:** Table of experiments.

S.N.	OBSERVATI-ON TIME	SLICE NO.	PIG NO.	EXP. PROBES	POLA-RTY	CONFIGUR-ATION	EXP. STEPS
1	24H	G	P2	2 probes	NA	Fig 2A	Cryo only–control (13min)
2	24H	C	P2	1 probe as cathode, 1 probe as anode	+/-	Fig 2E	Freeze (13min) →EL (13min,50mA)
3	24H	B	P2	1 pad as anode, 2 probes as **cathode**	-	Fig 2B	Freeze (13min) →EL (13min,50mA)
4	24H	A	P2	1 pad as cathode, 2 probes as **anode**	+	Fig 2C	Freeze (13min) →EL (13min,50mA)
5	48H	U	P4	2 probes	NA	Fig 2A	Cryo only–control (13min)
6	48H	J	P4	**2 probes as cathode** 2 probe as anode	-	Fig 2D	Freeze (13min) →EL (13min,50mA)
7	48H	I	P4	2 probes as cathode **2 probe as anode**	+	Fig 2D	Freeze (13min) →EL (13min,50mA)
8	24H	O	P5	1 probe as cathode **1 probe as anode**	+	Fig 2F	Freeze (13min) →EL (13min,50mA)
9	24H	P	P5	**1 probe as cathode** 1 probe as anode	-	Fig 2F	Freeze (13min) →EL (13min,50mA)

**Fig 3:**
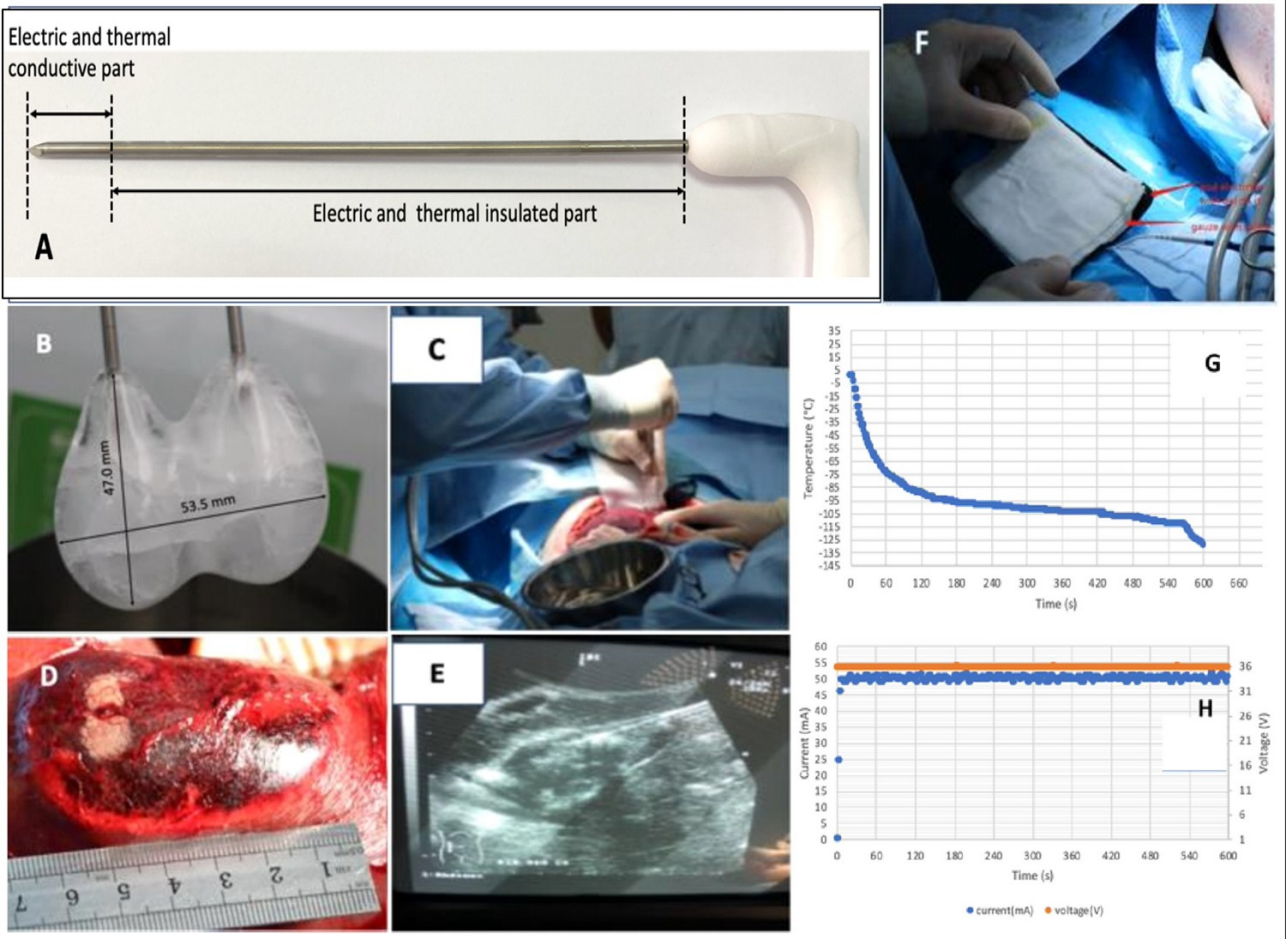
Cryoelectrolysis procedure details.

A photograph of the cryoelectrolysis probe used in all the experiments is shown in Fig 3A. When connected to the pressure regulator the probes act as a cryoablation probe and when connected to the power supply they act as an electrolysis electrode (Fig 1C). In these experiments, two cryoelectrolysis probes are inserted in parallel, they are inserted normal to the outer surface of the liver, to a depth of 15 mm from the outer surface. The distance between the probes is 25 mm. In order to hold the probes at the required distance, a holder, custom made of Plexiglass, is used as a spacer. The holder has two parallel drilled holes separated by 25 mm from each other, compatible with the 2.8 mm of the cryoelectrolysis probe. Fig 3B shows the ice balls which form when two parallel cryoprobes separated by 25 mm are immersed in water at room temperature. When used in the liver, the bottom of the spacer block was pushed against the liver lobe surface. Fig 3C is a photograph of a physician placing parallel cryoelectrolysis probes in the liver. A typical cryoelectrolysis experiment is as follows. After positioning the probe(s) in the liver through the spacer, freezing commenced. Freezing was generated using 3000 PSI Argon gas delivered at constant flow to both probes. The duration of freezing was determined from observation of the ice formation on the top surface of the liver (see Fig 3B). Freezing was terminated when the two large merging ice-balls clearly visible on the surface of the liver merged, e.g., Fig 3D. In most of the experiments this was at 13 minutes following the start of the freezing process. Therefore, we chose 13 minutes as the time for freezing in all the experiments, for consistency. The electrolysis generating DC power supply was activated at the instant freezing was stopped. Controlled electrolysis was delivered for 13 minutes (50 mA per cryoelectrolysis probe). We chose 13 minutes of electrolysis arbitrarily, for symmetry with the time of freezing. While 13 minutes of thawing is typical to conventional cryosurgery procedures, we had no guidance for choosing the time of electrolysis. For clinical benefit, it should not be longer than a second freeze-thaw cycle. The surface pad is shown in (Fig 1F). It is a commercial physiotherapy electrode (precoated with gel, Cholcom, Physiotherapy Electrode, Guangdong, China). A piece of gauze soaked with physiological saline was interposed between the surface pad and the pig skin, to serve as a reservoir for ions for the pad electrode and eliminate electrolytic damage to the skin. The entire cryoelectrolysis process was also monitored on ultrasound (Fig 3E). The cryoelectrolysis probes were removed at the end of electrolysis, and the insertion site was examined. If any bleeding was present after the procedure, a gentle pressure of a gauze for 5 minutes was able to stop and control the event.

### Measurements

Every experiment was monitored and recorded using ultrasound, video and photo. Freezing was delivered in a controlled way by maintaining a constant Argon gas pressure of 3000 psi and the pressure monitored. The electrolysis current was set at a constant 50 mA per cryoelectrolysis probe pair and the current monitored. Since the amount of electrolytic compounds depends only on the charge (current) delivered the relative location of the different polarity electrodes is immaterial to the treatment process. Only the amount of current delivered between pairs of electrodes determines the amount of electrolytic products delivered. The internal control circuit of the prototype cryoelectrolysis power supply is used for current, voltage and temperature monitoring. Typical measurements during a procedure are shown in Fig 3G for temperature at the tip of the probe and 3H for current through the probe.

### Histological examination

After the treatment, the pigs were kept alive for 24 hours or 48 hours, see Table 1. To remove the livers, the animals were euthanized with an overdose of 200 mg Propofol, followed by induction myorelaxant and pain relief as in the first experiment surgeries. The abdomen was opened removing the sutures. The vena cava was closed and then cut to produce total bleeding. The liver was then removed, placed on a table and each lesion was flushed with saline and the macroscopic appearance of the lesions identified and measured. The lesions were bread loafed along the cryoelectrolysis probes central path and soaked in 4% paraformaldehyde (PFA) (RNase free) for 72 hours until embedded in paraffine blocks. For microscopic analysis, 3 μm sections were cut from the samples and stained with Hematoxylin and Eosin (H&E). Micrographs were taken and digitized by digital slice scanner (Model: KF-PRO-005, KFBIO Co., Ltd., China).

## Results and discussion

In the following paragraphs, ablation will be evaluated by looking at macroscopic impression of the lesion and distinctive microscopic features of H&E stained slices. The focus of the analysis is comparing the histology of tissue treated with cryoablation with the histology of tissue treated with cryoelectrolytic ablation and highlight the differences.

**Fig 4:**
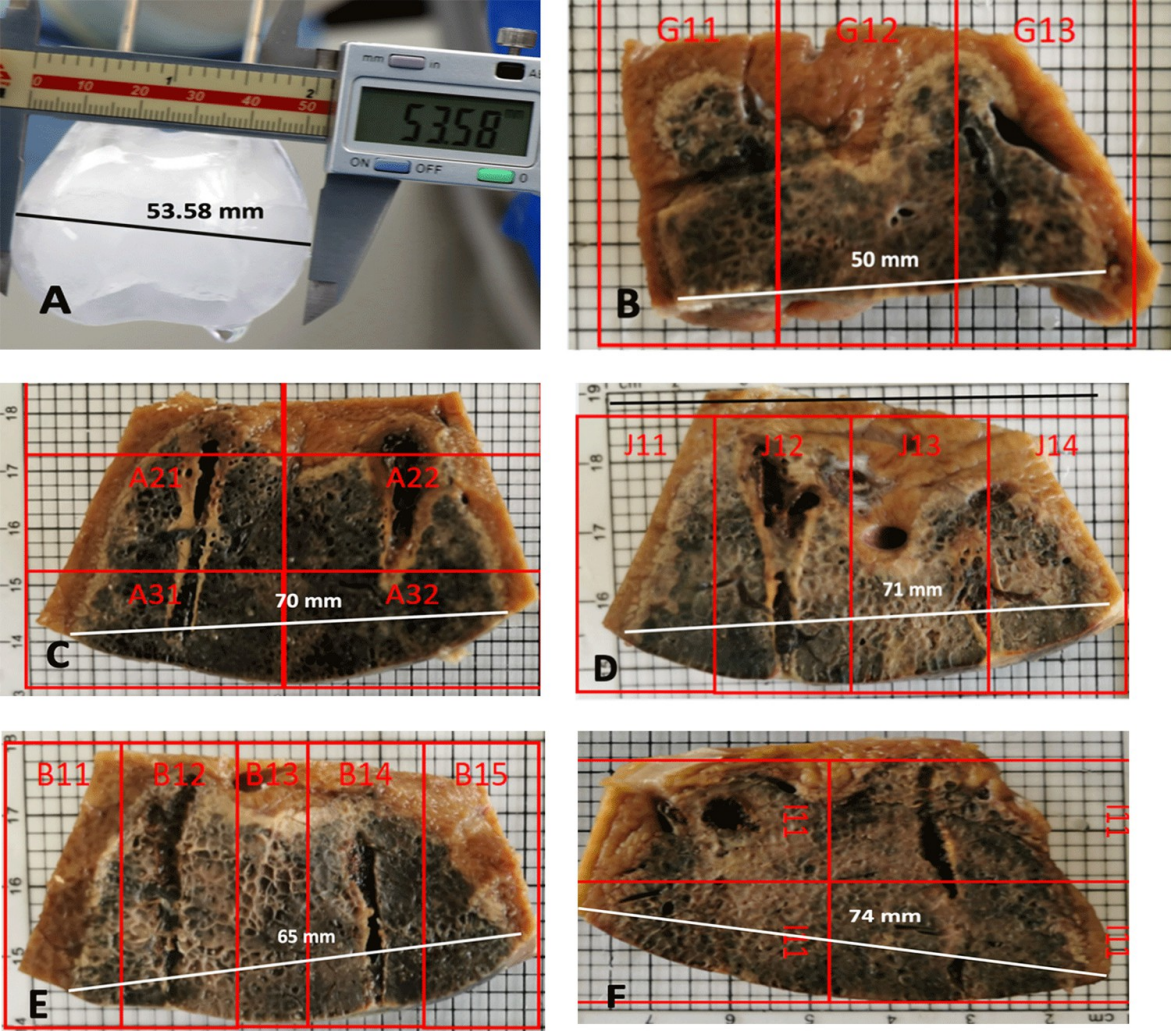
Macroscopic cross sections of liver treated with cryoablation and cryoelectrolysis following fixation.

Fig 4 shows macroscopic cross sections through liver tissue treated with cryoablation and cryoelectrolysis following fixation. The images were obtained from a bread loaf cut along the plane of the two cryoelectrolysis probes. Lighter margins surround the darker affected tissue. The length bars in each panel give the measured maximal width of the treated lesion. It should be emphasized that the number of experiments in this study is too low to be used for rigorous statistical analysis. Therefore the analysis afforded by this figure should be viewed as qualitative only.

Panel 4A shows the ice ball formed by immersing two cryoablation probes in water at room temperature. The maximal width here is 53.85 mm. This image is used as a control reference. Panel 4B shows a typical macroscopic appearance of liver tissue treated by cryoablation only, after fixation (configuration Fig 2A, experimental data Exp#1 & 5 in Table 1). In this figure, the width of the affected tissue after treatment with cryoablation only, is 50 mm. The measured dimension of the cryoablation treated liver is compared with the dimension of the ice ball in water in Panel 4A (Fig 4A). The smaller size of the ablation width in comparison to Fig 4A is because of the effect of blood flow in living tissue and because tissue survives the outer margin of the frozen lesion.

Fig 4C is from a cryoelectrolysis ablation with the configuration of Fig 2C and the treatment parameters in Table 1 (Exp. #4). Fig 4D is from a cryoelectrolysis ablation protocol with the configuration of Fig 2D and the treatment parameters in Table 1 (Exp. # 6 & 7). Fig 4E is from a cryoelectrolysis ablation with the configuration of Fig 2B and the treatment parameters in Table 1 (Exp. #3). Fig 4F is from a cryoelectrolysis ablation protocol with the configuration of Fig 2D and the treatment parameters in Table 1 (Exp. # 4).

Some observations are evident from Fig 4. It should be emphasized that the number of experiments is limited and therefore no valid statistical conclusions can be reached. Nevertheless, it is evident that the width of the region ablated with cryoelectrolysis is wider by about 20 mm from the width of the region ablated with cryoablation alone in all four different experiments, for the different configurations. It also appears that the extent of ablation is larger 48 hours after ablation than 24 hours after ablation. While not a statistically supported observation we nevertheless found that the extent of tissue affected by cryoelectrolytic ablation is always larger than the extent of tissue affected by cryoablation alone, in all the experiments without any exception; as illustrated by Fig 4. This suggest that electrolysis has a genuine effect–it increases the extent of the treated region over that treated by freezing alone. Obviously much more work needs to be done to quantify this effect statistically.

Both cryoablation and cryoelectrolysis resulted in massive necrosis of hepatocytes. Local hepatocyte apoptosis was observed at the edge of the necrotic area after cryoablation and cryoelectrolysis.

**Fig 5:**
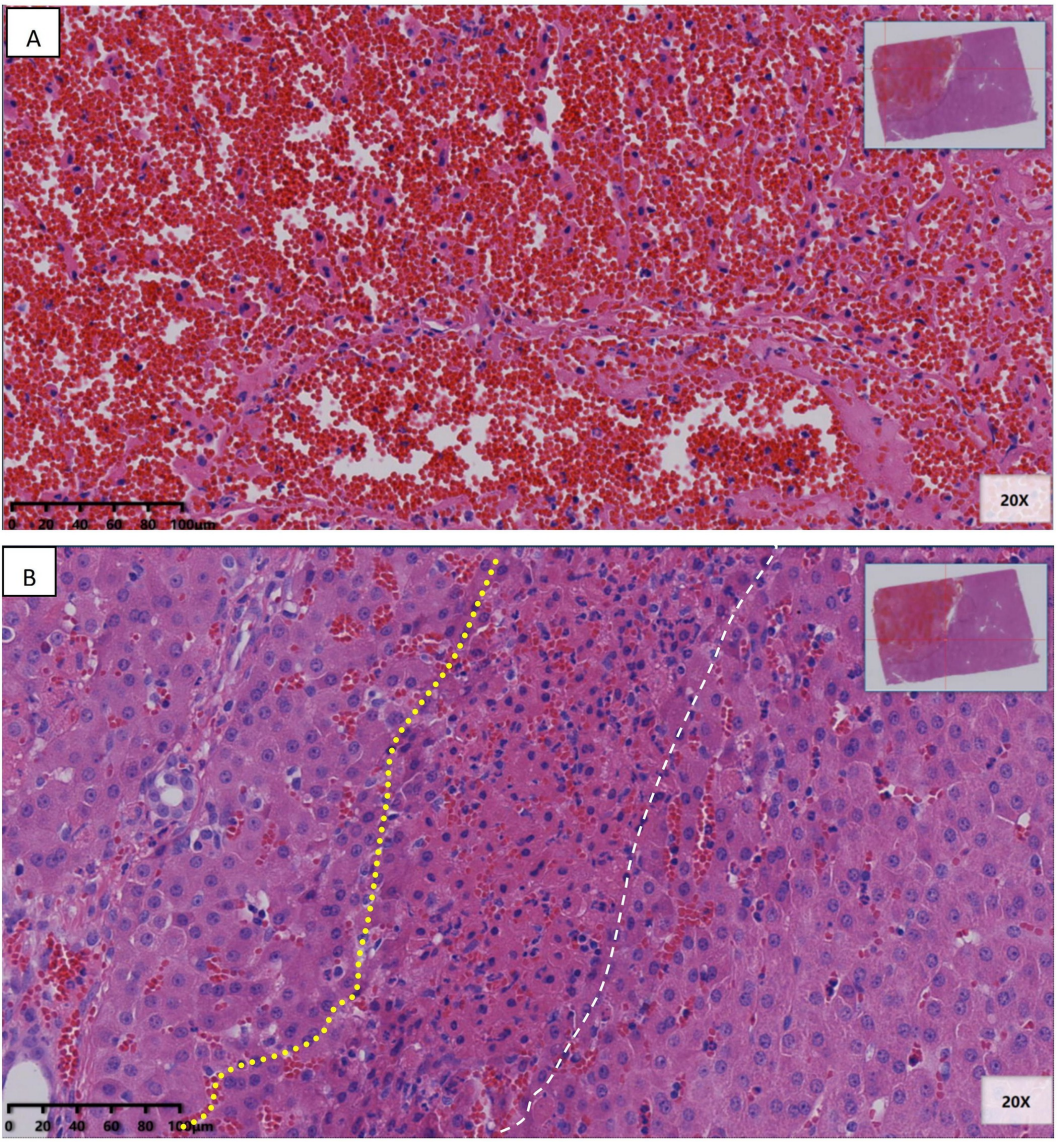
Liver margin and liver core treated with cryoablation only.

Fig 5 shows liver margin and liver core treated with cryoablation only, in configuration of Fig 2A and taken from experiment #1. They are typical to the outcome of cryoablation. Coagulation necrosis and hemorrhage of hepatocytes were observed in the center of the cryoablation necrotic area (Fig 5A). At the margin of the cryoablation necrotic area, coagulation necrosis of hepatocytes was observed, showing the retention of the original hepatocyte contour, nuclear pyknosis, and nuclear membrane integrity. In addition, cell survival was observed around the portal area, and there was a large area of cell survival on the dorsal side of the large vessels (Fig 5B).

**Fig 6:**
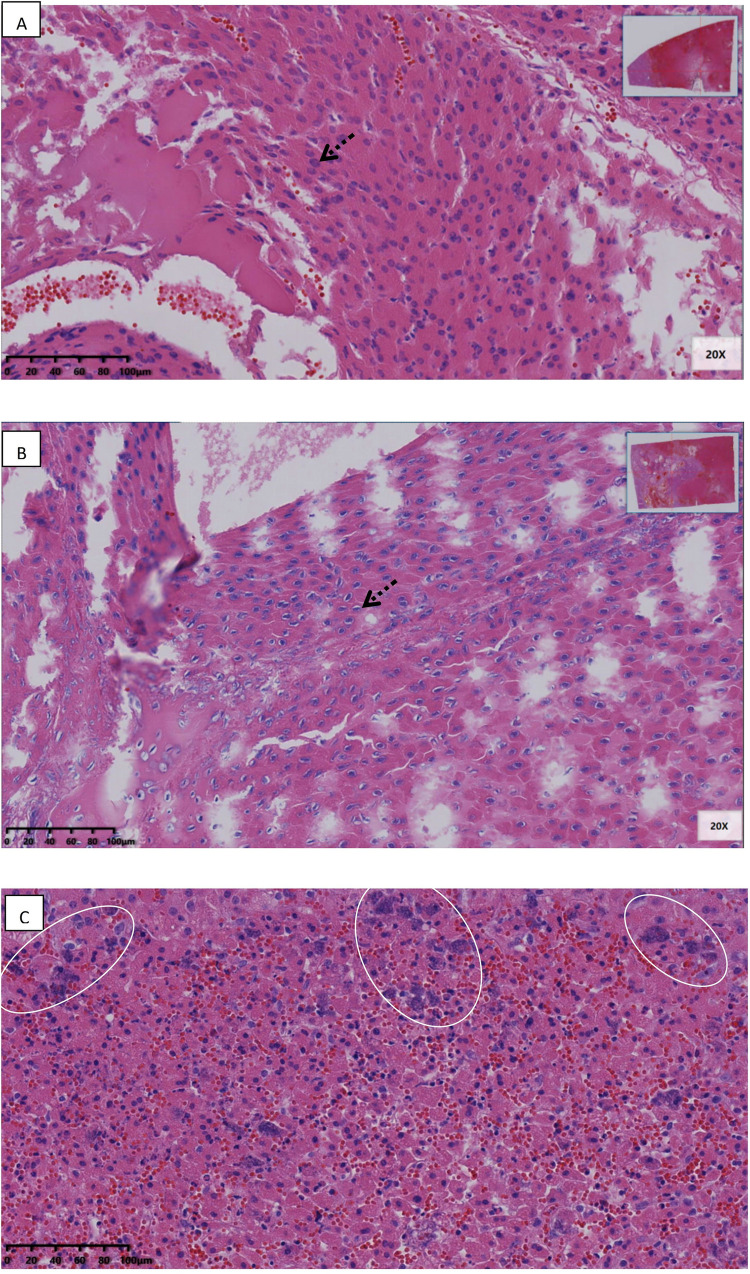
Liver margin and liver core treated with cryoelectrolysis.

Fig 6 shows liver margin and liver core treated with cryoelectrolysis, in configuration of Fig 2A and taken from Exp. #6 & 7. In addition to necrosis and bleeding, edema was also severe in the center of the cryoelectrolysis necrotic area. Besides, necrosis cells were contracted around the anode, intercellular spaces and hepatic sinusoids were dilated, and edema fluid was gathered (Fig 6A). The cells around the cathode showed typical changes of electrolysis cell nuclei, including enlarged nuclei and radial arrangement of nuclear chromatin (Fig 6B). In addition, several cyst-like structures with accumulation of exudate or blood of different sizes were observed around the electrode. In the cryoelectrolysis treated margin area, the necrosis structure of hepatocytes was completely destroyed, including cell rupture, nuclear membrane rupture, and nuclear contents mixed with cytoplasm to form basophilic mass like structure (Fig 6C).

In summary, this is additional experimental data which provides evidence that electrolysis delivered from the cryoelectrolysis probe delivered during the thawing stage of the ablation procedure has an effect on the treated tissue. This is supported by the following observations. First, histology shows that there is a substantial difference between the histological appearance of tissue treated with cryoelectrolysis at the anode and cathode from the appearance of tissue treated with cryoablation alone. Further support to the proof is the observation that the effect of electrolysis at the anode is different from that at the cathode. Particularly interesting is that there are vacuoles near the anode and liquid accumulation near the cathode. This is consistent with an electrochemical potential driven flow during electrolysis. The electrochemical potential drives fluid flow from the anode to the cathode, which can explain the accumulation of the edema at the cathode side and the vacuoles at the anode side. Also, at the anode the tissue becomes acidic and at the cathode it becomes basic. This difference in pH explains why cell death is different at the anode from the cathode, and is further evidence that electrolysis delivered during the thawing stage affects the cryoelectrolysis treated tissue. The fact that electrolysis changes the pH of tissue may play an important additional role in the treatment of cancer with cryoelectrolysis. It is known that cancer cells viability is affected by the pH of their environment, e.g. [35]. It remains to be checked but it is possible that the changes in pH during cryoelectrolysis may destroy cancer cells residues around the treated tissue. As discussed in [35], reducing the acidity around cancer cells inhibits their ability to proliferate–suggesting that cryoelectrolysis with cathodes may add additional benefits.

## Conclusions

This is a limited study on the effect of electrolysis delivered through a cryoelectrolysis probe during the thawing stage of a cryoablation procedure. The qualitative results show that the extent of tissue affected by cryoelectrolytic ablation is larger than that of tissue affected by cryoablation alone. Furthermore, cell death from cryoelectrolysis is different from cell death by cryoablation alone. It was interesting to observe that the effect of electrolysis at the anode on cell death is different from the effect of electrolysis on cell death at the cathode. The results of this first order investigation confirm that electrolysis has an effect on cell ablation by freezing and supports further research in the field.

## References

[1] Gage A., “History,” in *Cryosurgery: Mechanisms and Applications*, Lucas L., Ed. Paris: Int. Inst. Refrigeration, 1995, p. 140.

[2] Baust J. G., Gage A. A., and Baust J. M., “Principles of cryoablation,” in *Dermatological Cryosurgery and Cryotherapy*, 2016, pp. 9–16.

[3] Arnott J., *On the Treatment of Cancer by the Regulated Application of an Anesthetic Temperature*. London: Churchill, 1850.

[4] Rubinsky B. and Shitzer A., “Analysis of a Stefan-Like Problem in a Biological Tissue Around a Cryosurgical Probe,” *ASME Trans*. *J*. *Heat Transf*., vol. 98, pp. 514–519, 1976.

[5] Gage A. A. and Baust J., “Mechanisms of tissue injury in cryosurgery,” *Cryobiology*, vol. 37, no. 3, pp. 171–186, 1998. doi: 10.1006/cryo.1998.2115 9787063

[6] Rubinsky B., “Cryosurgery,” *Annu*. *Rev*. *Biomed*. *Eng*., vol. 2, pp. 157–187, 2000. doi: 10.1146/annurev.bioeng.2.1.157 11701510

[7] Gage A. A., Baust J. M., and Baust J. G., “Experimental cryosurgery investigations in vivo,” *Cryobiology*, vol. 59, no. 3, pp. 229–243, 2009. doi: 10.1016/j.cryobiol.2009.10.001 19833119 PMC2821701

[8] Baust J. G. et al., “Re-purposing cryoablation: a combinatorial ‘therapy’ for the destruction of tissue,” *Prostate Cancer Prostatic Dis*., vol. 18, no. 2, pp. 87–95, 2015. doi: 10.1038/pcan.2014.54 25622539

[9] Baust J. G., Snyder K. K., Santucci K. L., Robilotto A. T., Van Buskirk R. G., and Baust J. M., “Cryoablation: physical and molecular basis with putative immunological consequences,” *International Journal of Hyperthermia*, vol. 36, no. sup1. pp. 10–16, 2019. doi: 10.1080/02656736.2019.1647355 31795837 PMC6897311

[10] Rubinsky B., “Cryosurgery imaging with ultrasound,” *Mech*. *Eng*., vol. 108, no. 1, pp. 48–52, 1986.

[11] Onik G., Cooper C., Goldberg H. I., Moss A. A., Rubinsky B., and Christianson M., “Ultrasonic characteristics of frozen liver,” *Cryobiology*, vol. 21, no. 3, pp. 321–328, 1984. doi: 10.1016/0011-2240(84)90327-4 6734242

[12] Pease G. R., Wong S. T. S., Roos M. S., and Rubinsky B., “Mr image‐guided control of cryosurgery,” *J*. *Magn*. *Reson*. *Imaging*, vol. 5, no. 6, pp. 753–760, 1995. doi: 10.1002/jmri.1880050623 8748498

[13] Edd J. F., Ivorra A., Horowitz L., and Rubinsky B., “Imaging cryosurgery with EIT: tracking the ice front and post-thaw tissue viability.,” *Physiol*. *Meas*., vol. 29, no. 8, pp. 899–912, Aug. 2008. doi: 10.1088/0967-3334/29/8/004 18603669 PMC2746765

[14] D. Sohn RL; Carlin AM; Steffes C; Tyburski JG; Wilson RF; Littrup PJ; Weaver, “The extent of cryosurgery increases the complication rate after hepatic cryoablation,” *Am*. *Surg*., vol. 69, no. 4, pp. 317–322, 2003. 12716090

[15] Nilsson E., Berendson J., and Fontes E., “Impact of chlorine and acidification in the electrochemical treatment of tumours,” *J*. *Appl*. *Electrochem*., vol. 30, no. 12, pp. 1321–1333, 2000.

[16] Perkons N. et al., “Electrolytic ablation enables cancer cell targeting through pH modulation,” *Commun*. *Biol*., vol. 1, no. 48, p. doi: 10.1038/s42003-018-0047-1, 2018. 30271931 PMC6123816

[17] Amory R., *A treatise on electrolysis and its therapeutical and surgical treatment in disease*. New York: William Woof &Co., 1886.

[18] Gravante G. et al., “Experimental application of electrolysis in the treatment of liver and pancreatic tumours: Principles, preclinical and clinical observations and future perspectives,” *Surg*. *Oncol*., vol. 20, no. 2, pp. 106–120, 2011. doi: 10.1016/j.suronc.2009.12.002 20045634

[19] von Euler H., Nilsson E., Olsson J. M., and Lagerstedt A. S., “Electrochemical treatment (EchT) effects in rat mammary and liver tissue. In vivo optimizing of a dose-planning model for EChT of tumours,” *Bioelectrochemistry*, vol. 54, no. 2, pp. 117–124, 2001. 11694391 10.1016/s1567-5394(01)00118-9

[20] Yoon D.-S. et al., “Introduction of electrochemical therapy (EChT) and application of EChT to the breast tumor,” *J*. *Breast Cancer*, vol. 10, no. 2, pp. 162–168, 2007.

[21] Turjanski P., Olaiz N., Abou-Adal R., Suarez C., Risk M., and Marshall G., “pH front tracking in the electrochemical treatment (EChT) of tumors: Experiments and simulations,” *Electrochim*. *Acta*, vol. 54, no. 26, pp. 6199–6206, 2009.

[22] von Euler H., Strahle K., and Yongqing G., “Cell proliferation and apoptosis in rat mammary cancer after electrochemical treatment (EChT),” *Bioelectrochemistry*, vol. 62, no. 1, pp. 57–65, 2004. doi: 10.1016/j.bioelechem.2003.10.008 14990326 PMC7129577

[23] Nilsson E. et al., “Electrochemical treatment of tumours,” *Bioelectrochemistry and Bioenergetics*, vol. 51, no. 1. pp. 1–11, 2000. doi: 10.1016/s0302-4598(99)00073-2 10790774

[24] Phillips M., Raju N., Rubinsky L., and Rubinsky B., “Modulating electrolytic tissue ablation with reversible electroporation pulses,” *Technology*, vol. 3, no. 1, pp. 45–53, 2015.

[25] Quinn P. J., “A lipid-phase separation model of low-temperature damage to biological membranes,” *Cryobiology*, vol. 22, no. 2, pp. 128–146, 1985. doi: 10.1016/0011-2240(85)90167-1 3920005

[26] Willem H. S. Wolkers F., Oldenhof Harriëtte, Tang Fengrui, Han Jiale, Bigalk Judith, “Factors Affecting the Membrane Permeability Barrier Function of Cells during Preservation Technologies.,” *Langmuir*, vol. 35, no. 23, pp. 7520–7528, 2019. doi: 10.1021/acs.langmuir.8b02852 30501184

[27] Mardones G. and Gonzalez A., “Selective plasma membrane permeabilization by freeze-thawing and immunofluorescence epitope access to determine the topology of intracellular membrane proteins,” *J*. *Immunol*. *Methods*, vol. 275, no. 1–2, pp. 169–177, 2003. doi: 10.1016/s0022-1759(03)00015-2 12667681

[28] Hjouj M., Khrishnan H., and Rubinsky B., “Cryoelectrolysis for treatment of atrial fibrillation: A first order feasibility study,” *CryoLetters*, vol. 38, no. 6, pp. 428–433, 2017. 29734438

[29] Manuel T. J., Munnangi P., and Rubinsky B., “An electrochemistry study of cryoelectrolysis in frozen physiological saline,” *IEEE Trans*. *Biomed*. *Eng*., vol. 64, no. 7, pp. 1654–1659, 2017. doi: 10.1109/TBME.2016.2627402 28113196

[30] Lugnani F., Macchioro M., and Rubinsky B., “Cryoelectrolysis-electrolytic processes in a frozen physiological saline medium,” *PeerJ*, vol. 2017, no. 1, pp. 1–14, 2017. 10.7717/peerj.2810 PMC5244893 28123904

[31] Rubinsky B. and Cravalho E. G., “Analysis of the temperature distribution during the thawing of a frozen biological organ.,” *AIChE Symp*. *Ser*., vol. 75, pp. 81–88, 1979.

[32] Lugnani F. et al., “A vivens ex vivo study on the synergistic effect of electrolysis and freezing on the cell nucleus,” *PLoS One*, vol. 10, no. 12, p. e0145133, Dec. 2015. doi: 10.1371/journal.pone.0145133 26695185 PMC4687922

[33] Lugnani F. et al., “Cryoelectrolysis; an acute case study in the pig liver,” *Cryobiology*, vol. 78, pp. 110–114, 2017. doi: 10.1016/j.cryobiol.2017.08.001 28782504

[34] Erinjeri J. P. and Clark T. W. I., “Cryoablation: Mechanism of action and devices,” *J*. *Vasc*. *Interv*. *Radiol*., vol. 8, pp. S187–S191, 2010. doi: 10.1016/j.jvir.2009.12.403 20656228 PMC6661161

[35] S. N. B. and Nazanin Rohani F. B. G., Hao Liangliang, Alexis Maria S., Joughin Brian A., Krismer Konstantin, Moufarrej Mira N., Soltis Anthony R., Lauffenburger Douglas A., Yaffe Michael B., Burge Christopher B., “Acidification of Tumor at Stromal Boundaries Drives Transcriptome Alterations Associated with Aggressive Phenotypes,” *Cancer Res*., vol. doi: 10.1158/0008-5472.CAN-18-1604, 2019. 30755444 PMC6467770

